# Complications as indicators of quality assurance after 401 consecutive colorectal cancer resections: the importance of surgeon volume in developing colorectal cancer units in India

**DOI:** 10.1186/1477-7819-10-15

**Published:** 2012-01-18

**Authors:** Guruprasad S Shetty, Yashodhan D Bodhankar, Sachin Ingle, Rohan G Thakkar, Mahesh Goel, Parul J Shukla, Shailesh V Shrikhande

**Affiliations:** 1 Department of Gastrointestinal Surgical Oncology, Tata Memorial Centre, Mumbai, India

**Keywords:** Colorectal cancer, Rectal Cancer, Complications, Surgery, Low anterior resection, Abdominoperineal resection, Hemicolectomy, Colectomy

## Abstract

**Background:**

The low incidence of colorectal cancer in India, coupled with absence of specialized units, contribute to lack of relevant data arising from the subcontinent. We evaluated the data of the senior author to better define the requirements that would enable development of specialized units in a country where colorectal cancer burden is increasing.

**Methods:**

We retrospectively analyzed data of 401 consecutive colorectal resections from a prospective database of the senior author. In addition to patient demographics and types of resections, perioperative data like intraoperative blood loss, duration of surgery, complications, re-operation rates and hospital stay were recorded and analyzed.

**Results:**

The median age was 52 years (10-86 years). 279 were males and 122 were females. The average duration of surgery was 220.32 minutes (range 50 - 480 min). The overall complication rate was 12.2% (49/401) with a 1.2% (5/401) mortality rate. The patients having complications had an increase in their median hospital stay (from 10.5 days to 23.4 days) and the re-operation rate in them was 51%. The major complications were anastomotic leaks (2.5%) and stoma related complications (2.7%).

**Conclusions:**

This largest ever series from India compares favorably with global standards. In a nation where colorectal cancer is on the rise, it is imperative that high volume centers develop specialized units to train future specialist colorectal surgeons. This would ensure improved quality assurance and delivery of health care even to outreach, low volume centers.

## Background

Rectal cancer in India is more common than colon cancer (colon cancer rates range from 3.7 to 0.7/100,000 among men and 3 to 0.4/100,000 among women whereas rectal cancer rates range from 5.5 to 1.6/100,000 among men and 2.8 to 0/100,000 among women) and trends show a high incidence among young Indians [1], a finding that can neither be explained by heredity nor traditional diet. This high incidence in younger patients makes it imperative that colorectal cancer management evolves in India and departments are expected to develop units specialized in multidisciplinary management of colorectal cancer to face future challenges.

However prior to development of colorectal cancer surgery as a specialty, it would be necessary to generate data from the Indian subcontinent. This need is hampered on account of two reasons - the incidence of colorectal cancer is very low compared to the West [1] and secondly there are very few teams performing specialized colorectal cancer surgery. Both these reasons are important in isolation but are also interlinked. Our group previously published the first large series from India on outcomes after double stapling technique for low rectal cancer [2].

In this paper, we analyzed the peri-operative outcomes following 401 consecutive colorectal resections by a single surgeon.

## Materials and methods

Between June 2002 and May 2009, the data of 401 patients who underwent surgery (by the senior author) for colorectal cancer were recorded in a prospective database. Furthermore, medical records were also examined to obtain patient demographics (age and sex), the type of surgeries performed, operative time, blood loss, postoperative hospital stay, perioperative morbidity and mortality etc. All patients had histologically proven adenocarcinoma of the colon or of the rectum. As part of a hospital protocol, all patients were meticulously evaluated preoperatively, to assess their general, nutritional and cardio-respiratory status. Bowel preparation was carried out preoperatively with polyethylene glycol the day before surgery in all cases. The type of surgeries performed were hemicolectomy, anterior resection (AR), abdominoperineal resection (APR), stoma related surgeries and other colectomies like subtotal colectomy, completion colectomy, sigmoid colectomy, total colectomy and proctocolectomy (others included ileotransverse bypass, resection & anastomosis of small bowel for obstructions, wedge resection of large bowel, stricturoplasty, reversal of Hartmann's procedure). Postoperative care included antibiotics (Amoxycillin with clavulinic acid 1.2 g 8 hourly) for 5 days, with patients being fed orally as soon as bowel sounds resumed. Drains were removed once bowel sounds returned and if there was no suspicion/evidence of an anastomotic leak. Complications recorded were bleeding, anastomotic leaks, wound related complications, intestinal obstruction, stoma related complications (i.e. sinking of stoma, stomal necrosis). Other complications included conditions like myocardial infarctions, deep vein thrombosis and respiratory complications. Conventional indicators for discharge were patients being clinically asymptomatic, fully ambulatory and tolerating a full diet. Any deviation from these indicators resulted in the patient being considered for longer hospital stay.

### Statistical analysis

Data maintenance and statistical analysis was performed by SPSS 14 software.

## Results

### Overall patient data

To compare the overall cohort, as well as the patients who developed complications, the data has been tabulated in the Table 1.

**Table 1:** Demographics & operative parameters.

Demographics	Overall	Complicated
n	401	49

Median age (years)	52 (10-86)	67.5 (18-72)

Male: Female	279:122	36:13

Hospital Stay (days)	10.52 (1-52)	23.4 (6-77)

Operative time (minutes)	220.32 (50-480)	224.33 (50-450)

Blood loss (ml)	418 (50-3000)	412.42 (50-1200)

The number of patients operated and the data recorded each year according to the type of surgery between June 2002 and May 2009 is provided in Table 2.

**Table 2:** Number of patients operated and the data recorded each year

Year	Right colectomies	AR	APR	Other colectomies	Stoma related surgeries	Others	Total
2002	3	8	4	1	4	2	22

2003	5	24	20	2	7	2	60

2004	7	20	17	10	10	3	67

2006	8	23	17	9	8	3	68

2007	11	26	14	9	12	3	75

2008	20	30	12	11	10	3	86

2009	3	11	4	1	3	1	23

Total	57	142	88	43	54	17	401

**N.B**. data was only captured until May 2009

### Data of patients who developed complications

The overall complication rate was 12.2% (49/401). The complication rate in 2002, '03, '04, '06, '07, '08 and '09 was 13.6%, 8.3%, 7.4%, 11.7%, 10.7%, 17.4% and 21.7% respectively. The year wise complication rate with the distribution of various complications every year is provided in Table 3.

**Table 3:** The distribution of various complications every year

Complications	2002	2003	2004	2006	2007	2008	2009	Overall
Total operated cases	22	60	67	68	75	86	23	401

Anastomotic Leaks	1	2	3	0	2	1	1	10 (2.5%)

Intestinal Obstruction	1	2	0	1	0	0	0	4 (1%)

Stoma related	0	1	0	1	3	4	2	11 (2.7%)

Wound related	1	0	0	2	1	5	0	9 (2.2%)

Other related	0	0	1	4	2	3	2	12 (3%)

Unrelated	0	0	1	0	0	2	0	3 (0.7%)

Total	3	5	5	8	8	15	5	49

% complications	13.6	8.3	7.4	11.7	10.7	17.4	21.7	12.2%

The re-operation rate was 51% (25/49 patients). The details of re-operations are provided in Table 4.

**Table 4:** Details of the complications & re-operations

Complications	Total	Re-operations	Causes
Anastomotic Leaks	10 (2.5%)	8 (disconnections, proximal diversions)	2 colostomy closures, 6 post AR

Intestinal Obstruction	4 (1%)	4(adhesiolysis, bypasses)	3 post APR, 1 post Rt. Hemicolectomy

Stoma related	11 (2.7%)	7(refashioning)	5 retraction/sinking stomas, 2 discolouration of stomas

Wound related	9 (2.2%)	2 (resuturing)	1 burst abdomen, 1 wound infection secondary suturing done

Other related	12 (3%)	4 (re-resections, peritoneal lavages)	2 postoperative bowel ischemias, 1 pelvic hematoma, 1 acute abdomen

Unrelated	3 (0.7%)	0	-

Total	49 (12.2%)	25	-

There were 5 mortalities out of 401 patients (1.2%). The details are provided in Table 5.

**Table 5:** Details of Mortalities

**Sr.No**.	Age	Sex	Primary Site	TNM	Primary surgery	Comments	Cause of death	Remarks
1	70	Male	Rectum	T3N0M0	Low Anterior resection with covering colostomy	NA	Pulmonary complication requiring ventilatory support-never recovered.	Adequate preoperative assessment of pulmonary functions & excluding a focus of infection is now a routine in our set up.

2	63	Male	Splenic flexure of colon	T3N0M0	Left hemicolectomy with Hartmann's procedure	Emergency exploration for intestinal obstruction requiring on table bowel decompression due to massive bowel dilatation. Duration of surgery was over 4 hours.	Died with sepsis	Pre-existing sepsis, inadequate perioperative fluid resuscitation and long duration of emergency surgery contributed to the mortality.

3	54	Male	Splenic flexure of colon	T4N1M0	Left hemicolectomy and distal pancreatosplenectomy	Local recurrence with gastrocolic fistula. Exploratory laparotomy with distal gastrectomy with transverse colectomy and revision of pancreatic margin with gastrojejunostomy and colo-colic handsewn anastomosis.	Died with undiagnosed leak and poor nutrition	In a locally advanced malignancy with recurrence we had been aggressive in treating in the absence of metastases.

4	58	Male	Sigmoid	T4N1M0	Anterior resection of rectum	NA	Died after 30 hours due to Massive MI	Unforeseen cardiovascular complications occur despite adequate preoperative work up.

5	47	Male	Ascending colon	T3N2M0	Palliative Right Hemicolectomy	On POD 5th developed abdominal pain and severe dyspnea, shifted to ICU with metabolic acidosis and put on ventilator. He died on POD6.	Sepsis with multiorgan failure	Poor nutritional reserves add up to major surgical stress combined with septic complication

There is an increase in the hospital stay and a high rate of emergency re-operation in patients who developed complications. Otherwise, there were no major differences in the data of the patients who had complications compared to those who did not. The rest of the data with regards to age, blood loss and duration of surgery were all comparable between patients with and without complications.

## Discussion

The morbidity and mortality rates following resections for colorectal cancer are 17.7-35% and 3-6% respectively in some major studies so far [3-7]. Anastomotic leaks have been the most dreaded of all colorectal complications, leading to high rates of re-operation, stomas and even death. Anastomotic leak rates reported so far have been between 1.1-3.8% [[3,4] and [8]]. Our clinically relevant anastomotic leak rate was 2.5% (10/401).

Stoma related complications are another major source of morbidity in high volume colorectal surgery units. Complications like stomal necrosis, retraction, bleeding, stenosis, prolapse and hernia, amongst others, were commonly seen. The stoma related complications have been reported as high as 10-70% in some studies [[9,10] and [11]]. Our data showed a 2.7% stoma related complication rate (11/401). This rather low rate is most likely due to the fact that certain complications like skin excoriation, odor, leakage and soiling were not documented in the database and hence could not be evaluated in our study. There is a decreasing trend in complications initially till the year 2004. This perhaps coincided with the initial learning curve of a surgeon & as the volume of operative work increased the complication rate declined further (2002-2004) suggesting improved technical refinement that goes hand in hand with surgical experience and confidence. Later on (2006-2009) however, there is an upsurge in the rate of complications. This can be attributed to more number of complex procedures being undertaken in sicker patients. In addition, being a teaching institute the dependence on other team members and colleagues constitutes an integral part of the teaching process (for e.g. all permanent colostomies following abdomino-perineal resections for low rectal cancer were always performed by the senior author in the earlier years but this was not the case as the years have progressed). The resultant inter-surgeon variability probably added to the increased complication rate in the later part of the study. Improved documentation of stoma related complications might have been another factor to explain this increase in number.

The re-operation rate, in the group of patients who developed complications was 51% (25/49) (Table 4). This number is somewhat high considering that majority of modern day subacute obstructions and stoma complications do not require surgery and settle down with conservative measures. In the early years it is plausible that we were overtly aggressive in treating postoperative complications like intestinal obstruction, stomal retraction/sinking/necrosis, leading to a high rate of re-operations.

In our study, the length of hospital stay for the entire patient cohort (10.5 days) is less compared to the length of hospital stay for patients with complications (23.4 days). It indicates an obvious negative effect of postoperative complications on prolonging the hospital stay. In a vast country like India, patients are coming from far off places seeking specialized treatment at a tertiary referral center like ours. Because of logistic issues it is difficult to discharge patients "early" from the hospital and manage their minor postoperative problems on outpatient basis. The concept of medical economics has not yet seeped in the Indian health system. Insurance based health management is not as well established as in the West. So occupying hospital beds for a day or two more does not affect the treatment cost. The overall effect of all these factors has resulted in longer in-hospital stay than expected, even in those patients without complications.

The limitation of this study design is that complete records of all complications were possibly not retrieved in a retrospective study like this. Furthermore, finer details of minor wound infections, so common in colorectal surgery, were not recorded/evaluated in this study.

Despite the above mentioned limitations and observations, our morbidity and mortality rate of 12.2% and 1.2% respectively compares favorably with global standards [3-7].

In colorectal surgery, there is a trend towards improved outcomes for patients having their care provided in high volume hospitals [[12-14] and [15]]. Furthermore, it is likely that specialty training and experience has an important and strong impact on outcomes for patients with colorectal problems. It has been reported over 2 decades ago that high- volume providers (threshold of 40 colectomies) had a more important impact on outcomes than high-volume centers [16]. High-volume physicians have lower mortality rates than low-volume physicians. The ratio of the standardized mortality rate for patients of low-volume physicians to patients of high-volume physicians was 1.26, which was significant. Hospitals with volumes of 40 or fewer procedures had a standardized mortality rate of 8.3%, whereas hospitals with volumes higher than 40 had a standardized mortality rate of 5.9%. The ratio of mortality rates for low- to high-volume hospitals was 1.41, which was significant. Also, among patients of both high and low-volume physicians, mortality rates tended to flatten out after hospital volumes of 40. Thus, 40 procedures appear to be a threshold hospital volume for this data set. A landmark study from Sweden [17] highlighted the impact of a dedicated surgical training program wherein a marked reduction in stoma rates and local recurrence rates, along with improvement in long term survival, was seen in 447 patients. Another study reported reduced morbidity and mortality by 50% following specialized training [18].

It thus appears that specialization with high volumes is the key to improve outcomes after surgery for colorectal cancer.

**Figure 1:**
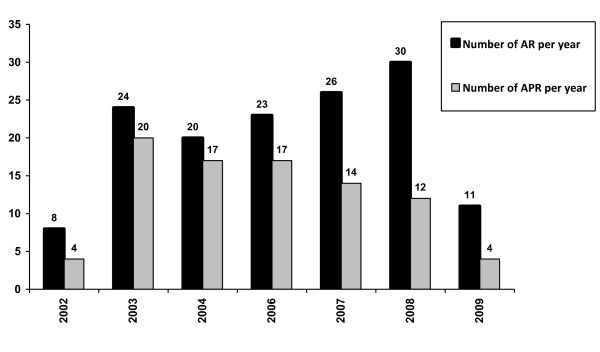
**Year wise comparative rates of AR: APR**.

In our study AR: APR rate was found to be declining over the years (Figure 1), resulting in fewer permanent stomas. AR: APR rate is also a useful proxy indicator of quality of care and presumably a marker of the increasingly experienced high-volume surgeon performing lower anastomoses [19]. Also the impact of neoadjuvant chemoradiation for locally advanced low lying rectal cancers resulted in offering sphincter conservation surgery to those who would have otherwise required abdomino-perineal resection [20].

It is pertinent to note that stomas may be an even bigger management problem in India than in the West, e.g. due to paucity of dedicated stoma clinics and nurses, high maintenance costs and availability of bags for the patients [21]. We have been well supported with an in house "ostomy clinic", which has adequate nursing staff trained in stoma care and a support group of "ostomates" who counsel and attend to the problems faced by the patients. This aspect needs to be factored in during the conception and development of specialized colorectal units.

Our current goals to further improve the standards of care revolve around emerging concepts like fast track surgery, reduced bowel preparation [In the absence of convincing data supporting bowel preparation, recently we have moved onto selective bowel preparation i.e. only for left sided colonic resections as the stool is more formed and hampers bowel handling with risk of spillage. So also we restrict bowel preparations only where anastomosis is planned, like in AR (anterior resection) and not in cases where end stoma is planned, as in APR (abdominoperineal resection)]. We have redefined our antibiotic policy (reduced to 3 days from 5 days), taken active steps to improve the scope of laparoscopic colorectal surgery [22], addressed issues related to early drain removal and ensured early feeding postoperatively [23]. We unfortunately could not identify any published data on major colorectal cancer resections from other centres within India and our study therefore assumes significance in encouraging other centres to develop their own databases and audit their work.

It is pertinent to note however, that specialized colorectal surgeons can also provide excellent results outside of high volume centers [24]. In the study by Ferenschild et al., well trained surgeons were able to achieve similar postoperative morbidity and mortality in rectal cancer patients with a comparable overall survival in a local community hospital. Also, quality assurance can be determined by an evaluation of perioperative parameters and complications. Previous studies have identified and rated indicators of high-quality perioperative care for patients undergoing surgery for colorectal cancer. The indicators can be used as quality performance measures and for quality-improvement programs [25].

In a recent paper, Billimoria et al. concluded that payers and oversight agencies are beginning to use structural characteristics such as surgeon training, experience, and volume as a basis for referral decisions [26]. They noted that majority of surgery residents are prolonging their training to gain additional experience. Thus, there is a need to understand specific factors which underlie the better outcomes for specialty-trained, experienced, high-volume surgeons. Extrapolating the above observations, despite a relatively low volume of 70-80 colorectal cases per year per surgeon, our data suggests that Tata Memorial Centre is favorably placed to provide a combination of high volumes and dedicated specialized training in colorectal cancer surgery in India. In an emerging nation like India, where the nature of tertiary healthcare is unable to penetrate to the far extents, it only seems essential that surgeons, favoring colorectal cancer surgery as a specialty, train in high volume specialized centres similar to ours to become trained high volume providers both for tertiary and even low volume centres. Further development of guidelines and quality measures addressing these factors can help to identify issues that inexperienced, non-specialty, low-volume surgeons can use to improve their own patient care [26].

## Conclusion

Our results compare favorably with data from other high volume centers. Extrapolating our experience we can safely assume that a high volume centre like ours is suitably geared to provide specialized training in colorectal cancer surgery. In the Indian subcontinent with overall low incidence of rectal cancer but relatively high incidence in the young, we need to accumulate and analyze data such as ours to develop specific guidelines to improve the quality of care for colorectal cancer. Such a step will further colorectal cancer surgery as a specialty thus enabling delivery of quality health care even to other low volume centers.

## List of Abbreviations

AR: Anterior Resection; APR: Abdomino-perineal Resection.

## Competing interests

The authors declare that they have no competing interests.

## Authors' contributions

Data collection and analysis, manuscript preparation, formatting, editing: SVS, GS, YB, SI, RT. Editing and critical review: MG, PJS, SVS. Concept, manuscript preparation, analysis and critical review: SVS. All authors read and approved the final manuscript.
